# Multiple hepatic and osseous focal lesions without splenomegaly and/or lymph nodes enlargement

**DOI:** 10.1186/s43066-023-00240-4

**Published:** 2023-02-13

**Authors:** Maged T. Elghannam, Moataz H. Hassanien, Yosry A. Ameen, Gamal M. ELattar, Ahmed A. ELRay, Mohammed D. ELtalkawy, Ahmed Y. Montasser

**Affiliations:** 1 Hepatogastroenterology Department, Theodor Bilharz Research Institute, Giza, Egypt; 2 Pathology Department, Theodor Bilharz Research Institute, Giza, Egypt

**Keywords:** Multiple hepatic focal lesions, Primary hepatic lymphoma, Immunohistochemical staining, Radiologic images, Chemotherapy

## Abstract

**Background:**

Hepatic involvement is a common extranodal manifestation of common and some rare hematologic malignancies. Although the imaging features of more common hepatic diseases such as hepatocellular carcinoma, metastases, and infection may overlap with those of hepatic hematologic malignancies, combining the imaging features with clinical manifestations and laboratory findings can facilitate correct diagnosis. Imaging has an important role in the diagnosis of hepatic focal lesions.

**Case presentation:**

A case presented with isolated multiple hepatic focal lesions without nodal or spleen enlargement diagnosed only by immunohistochemical study and turned out to be primary hepatic lymphoma (PHL). PHL is rare with roughly 100 described cases and accounts for less than 1% of all non-Hodgkin lymphomas. Osseous involvement adds more challenge to the diagnosis.

**Conclusion:**

Hepatologists must be aware of PHL as it may be confused with more common hepatic diseases, mainly multifocal HCC and/or hepatic metastasis.

## Background

Multiple hepatic focal lesions can be caused by hepatocellular carcinoma (HCC), metastasis, hematologic malignancy whether primary or secondary and infection, or abscess in patients taking chemotherapy [1]. Such a diagnosis is rarely considered by hepatologists. The aim of reporting such cases is to shed light and add more concern on diagnostic facilities and how to proceed to diagnose with stress on the role of imaging techniques and immunohistochemical study, appropriate diagnosis, and consequently management because surgery or liver-directed therapy (for HCC and metastasis) and antibiotic administration and drainage (for infection) may be obviated versus chemotherapy for hematologic malignancies. Here, we report a case of multiple hepatic and osseous focal lesions.

## Case presentation

Egyptian male patient is 52 years old. He is a shisha smoker until the year 2006. He is not a drug addict or has received any hormonal therapy before.

His past history has been significant only for tonsillectomy since 2006 and disc removal and plate and screw since 2014, after which he became a user of NSAIDs. Medically, he is known to have NIDDM on metformin XR 1 g BID and empagliflozin 25 mg tablet OD. Also, he is hypertensive on co-tareg 160/12.5 mg tablet. Recently, there is a history of COVID-19 infections during the last month.

**Fig. 1:**
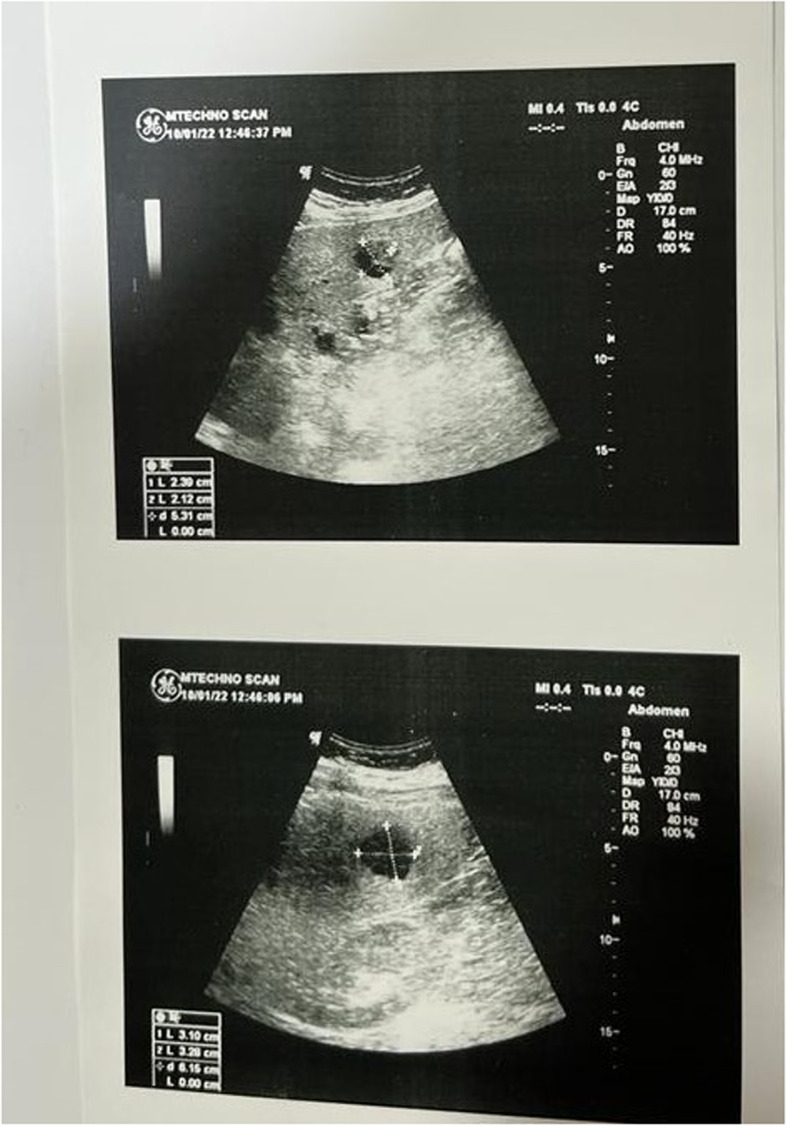
Abdominal ultrasonography. Multiple variable-sized hepatic complex cystic focal lesions

The present history started 2 months ago when he felt bilateral back pains. He sought medical advice, and his doctor asked for abdominal ultrasonography (Fig. 1). Surprisingly, he discovered multiple variable-sized hepatic complex cystic focal lesions; the largest at the RT lobe measures 3.1 × 3.2 cm and at Lt lobe measures 2.3 × 2.1 cm on top of the markedly enlarged diffuse fatty liver. His doctor asked for blood tests and abdominal CT. The laboratory tests were as follows: CBC: *HB* 13.6 g/dL (normal range 12.5–17.5); total leucocytic count 10.2 × 10^3^/mm^3^ (4–11), platelet count 251 × 10^3^ (150–450); *CRP* 199.4 mg/L (0–5), prothrombin time 11.7/10.6 s (8.2–13.1), concentration 86% (70–120), and *INR* 1.07 (0.9–1.27). *APTT* 29.2 s (23–40), total bilirubin 0.85 mg/dl (0.3–1.2), direct bilirubin 0.22 mg/dl (0–0.3), *ALT* 32 U/L (7–40), *AST* 36 U/l (0–34), serum albumin 4.7 g/dL (3.2–4.8), serum creatinine 0.67 mg/dL (0.7–1.3), and HBsAg and anti-HCV antibody were negative. Alfa-fetoprotein is 2.5 ng/dL (up to 8), *CEA* 1.6 ng/dL (up to 3), *CA* 19–9 2 U/ml (0–34), *LDH* 222 U/L (135–225), FBS 152 mg/dl (70–110), and 2 hpp 153 mg/dl (70–139).

Triphasic CT showed multiple hypodense hepatic focal lesions in both hepatic lobes; the largest one was 34 × 32 mm within segment VI with mild enhancement on post-contrast series suggestive of neoplastic lesions on top of markedly enlarged fatty liver.

A CT-guided biopsy was ordered and revealed a florid inflammatory reaction. For confirmation, a second guided biopsy was done by another doctor and revealed the same histologic diagnosis.

**Fig. 2:**
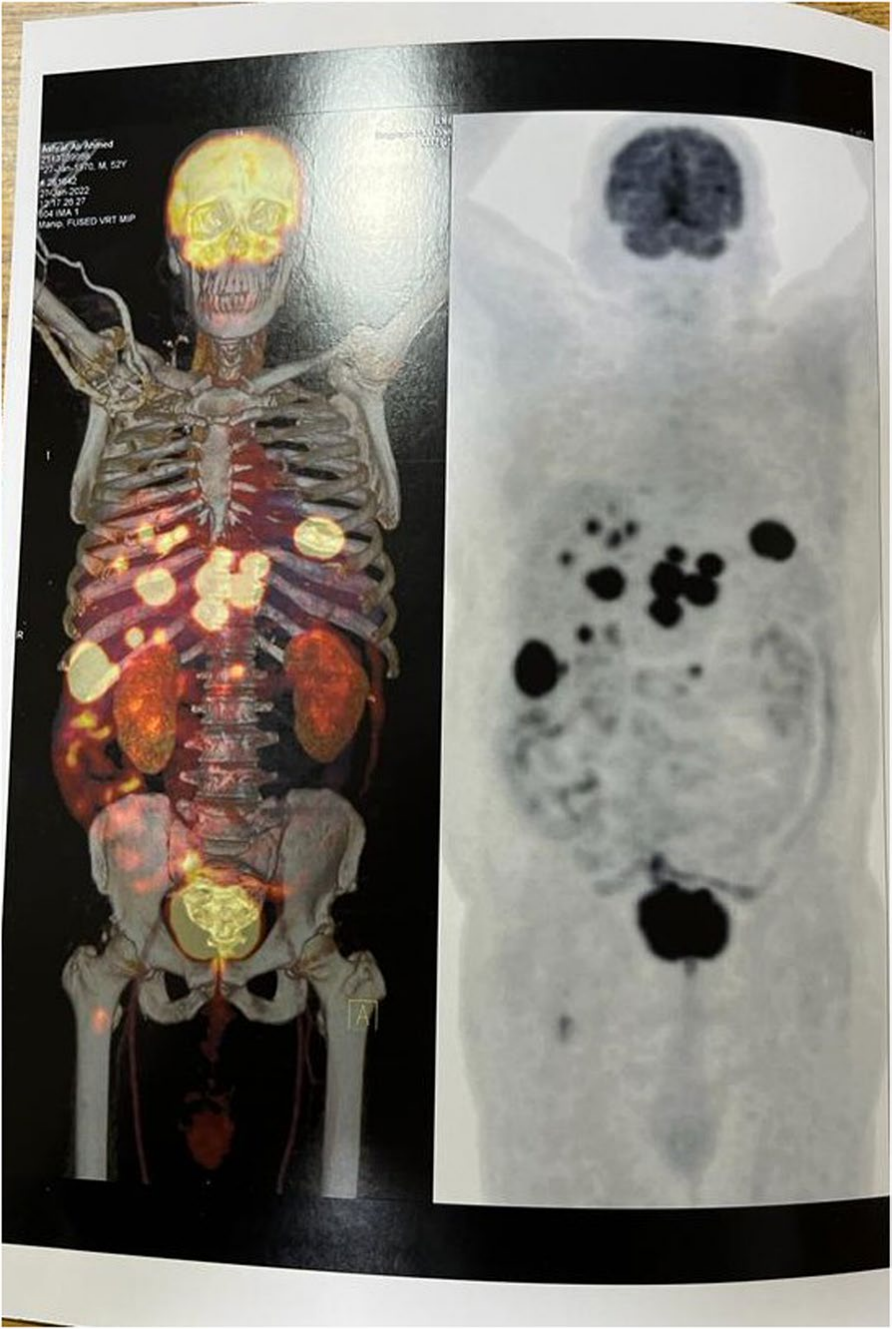
Whole-body PET-CT. Variable-sized metabolically active bilobar focal hepatic lesions in addition to active marrow-based osseous lesions at L1 vertebral body and proximal RT femoral shaft

The whole-body PET-CT examination was ordered and showed variable-sized metabolically active bilobar focal hepatic lesions. The largest and most active is seen at the RT lobe segment VI, measuring 5.5 cm in diameter. In addition, there are 2 metabolically active marrow-based osseous lesions in the L1 vertebral body and the proximal RT femoral shaft. The suggested provisional diagnosis includes lymphomas vs metastatic multifocal primary hepatic malignancy (Fig. 2).

**Fig. 3:**
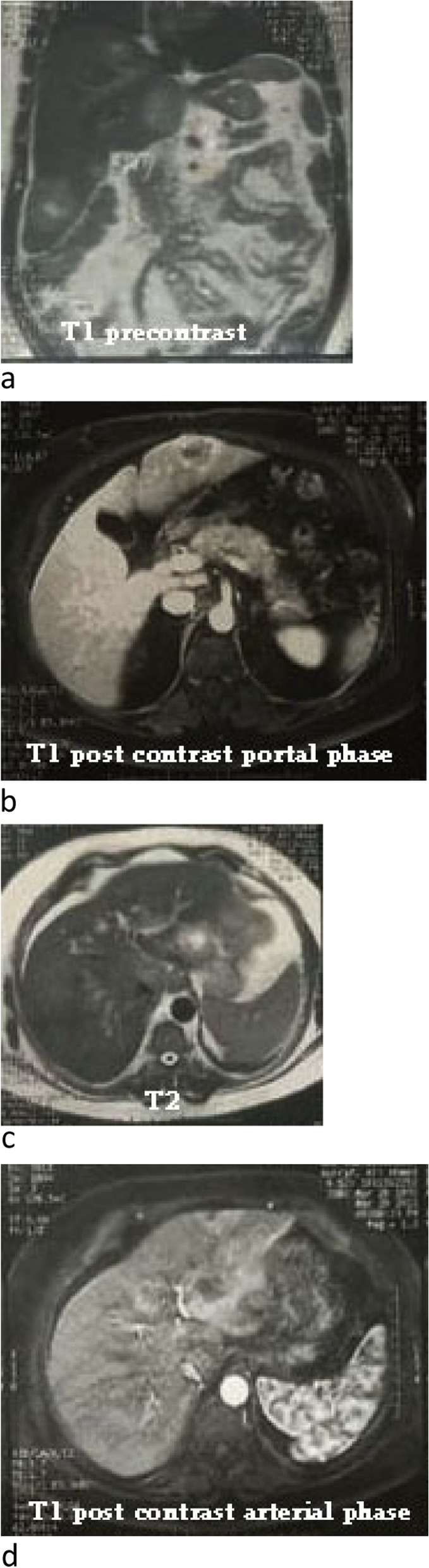
Abdominal MRI. Heterogeneous low T1 and higher T2 signal with some element of diffusion restriction along its periphery

The patient was referred to a consultant hepatologist. The physical examination was impressive only for hepatomegaly. He asked for dynamic MRI and immunohistochemical staining of the tissue biopsy. The dynamic MRI showed multiple hepatic bilobar mass lesions, the largest involving segment VI measuring 50 × 50 mm, in cross-sectional diameters. The lesions display heterogeneous low T1 and higher T2 signals with some elements of diffusion restriction along their periphery. The contrast uptake is seen in the arterial phase with washout thereafter. There is lower lumber transpedicular fixation. He considered metastatic vs multifocal HCC (Fig. 3).

**Fig. 4:**
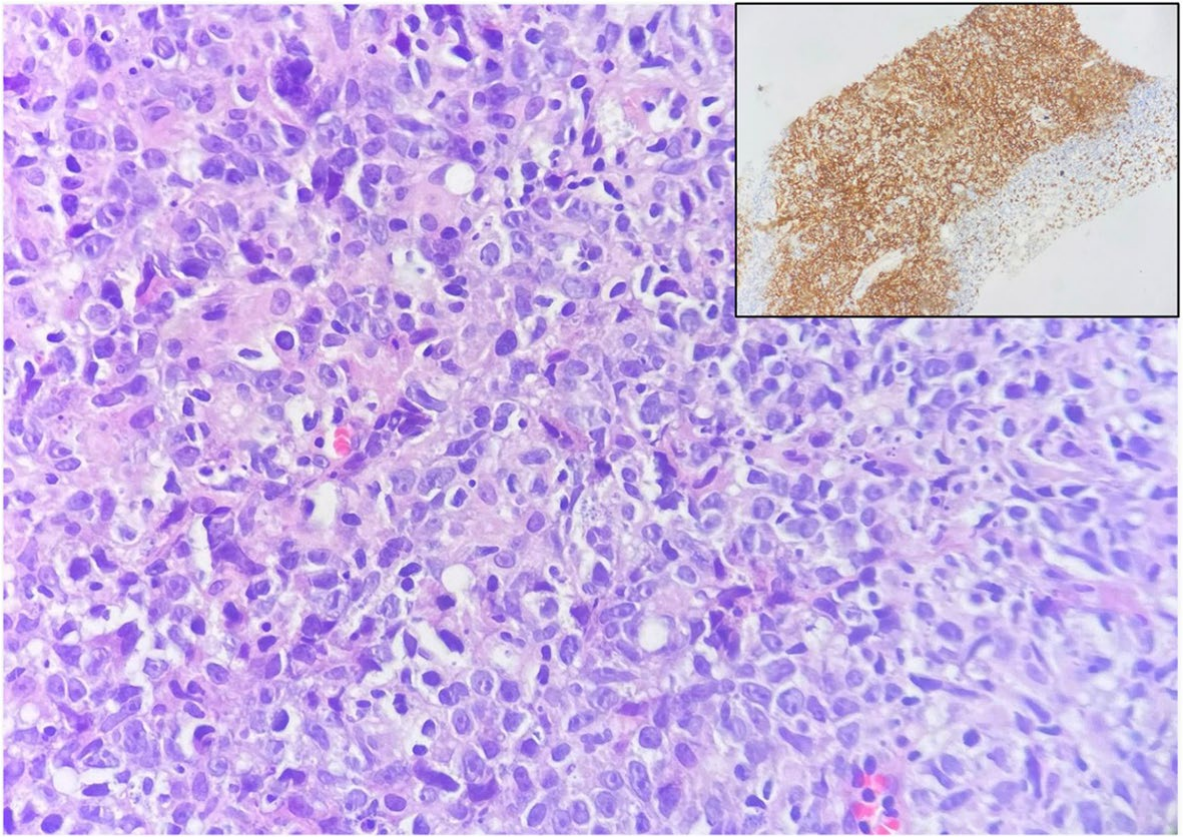
Histopathology and immunohistochemical staining. Diffuse sheets of large lymphoid cells with large irregular nuclei, focally showing prominent nucleoli and many nuclear debris (H&E × 400), diffusely positive for CD20 (inset, immunostain × 100)

**Fig. 5:**
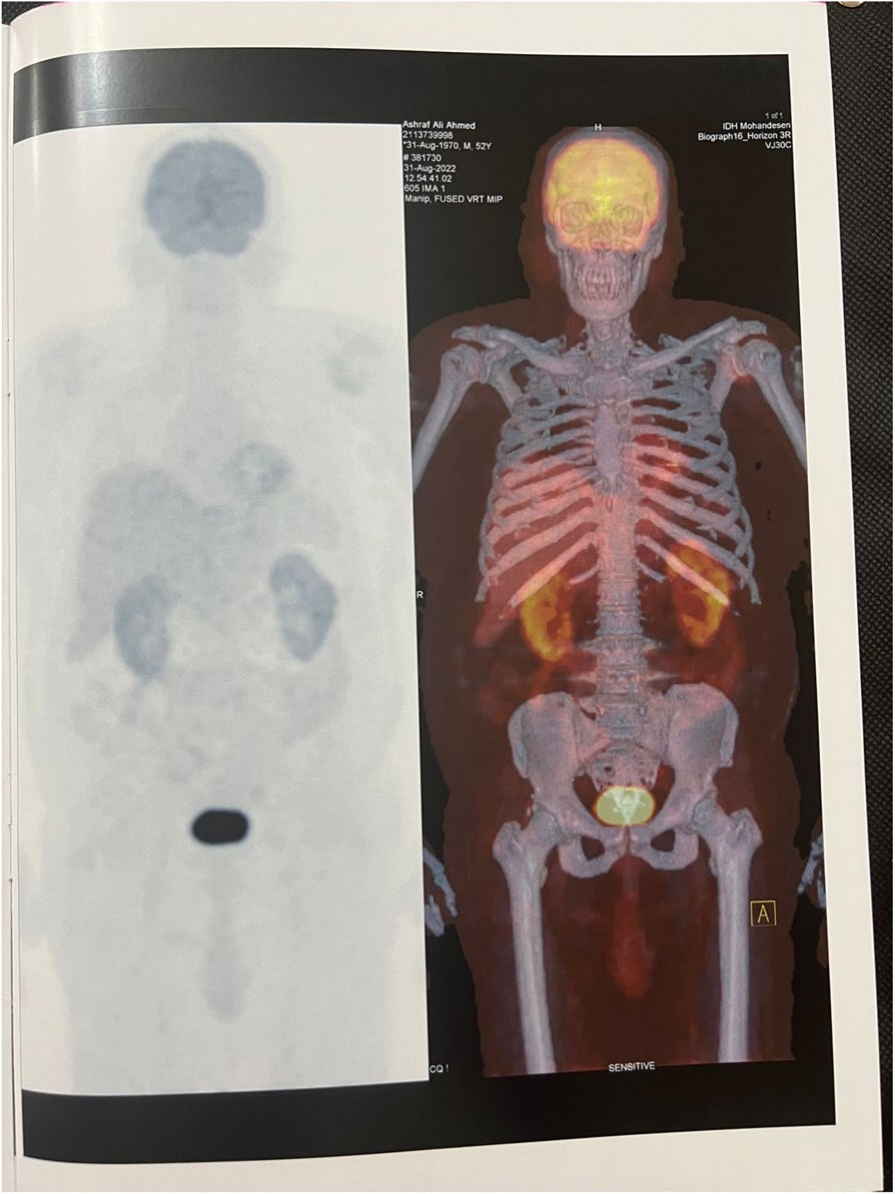
Posttreatment whole-body PET-CT. Morphological regression of the metabolically inert multiple hepatic focal lesions with no newly developed metabolically active lesions

The immunohistochemical sections were treated against CK-PanCK, CD10, Bcl6, Mum1, and Ki67. The results showed large neoplastic lymphoid cells which are strongly positive for CD20 and are positive for BCL6 and Mum1. The findings are consistent with large B-cell lymphoma and post germinal center origin (activated B-cell type) (Fig. 4). The patient was referred to an oncologist and started chemotherapy (R-CHOP) for 6 sessions, one session every 3 weeks. The fever and body aches disappear after the first session. Three months after, the chemotherapy sessions were fulfilled; a follow-up PET-CT was done and showed further morphological regression of the metabolically inert multiple hepatic focal lesions with no newly developed metabolically active lesions all over the body, indicating sustained complete metabolic disease remission (Fig. 5).

## Discussion

Malignant lymphomas, Hodgkin disease (HD), and non-Hodgkin lymphoma (NHL) account for approximately 5–6% of all malignancies [2]. Primary hepatic lymphoma (PHL) is defined as lymphoma that is confined to the liver and perihepatic nodal sites at patient presentation, without distant involvement [3]. PHL is rare with roughly 100 described cases and accounts for less than 1% of all non-Hodgkin lymphomas [1]. The incidence of PHL has increased in recent years, particularly in patients with human immunodeficiency virus (HIV) infection, predominantly because of immunosuppression. Pathologically, most cases of PHL are of B-cell lineage [4].

PHL cell infiltration of the liver with hepatomegaly is more common in NHL than in HD, with 16–43% of cases showing hepatic involvement [5]. More than 50% of patients with PHL present with right upper quadrant pain or jaundice. The B symptoms (systemic symptoms) of lymphoma, such as fever, body aches, and weight loss, are found in about one-third of patients with PHL [3]. PHL is commonly associated with viral hepatitis B and C and Epstein-Barr virus, but the pathophysiology of PHL is poorly understood [6].

Imaging has an important role in diagnosis of hepatic focal lesions. Lymphomatous involvement of the liver may manifest at imaging as a discrete focal liver mass or masses, diffuse infiltrating disease, or an ill-defined mass in the porta hepatis. The most common imaging manifestation of PHL is a solitary discrete lesion, which is seen in about 60% of cases. Multiple lesions are seen in 35–40% of patients [7], although one lesion is likely to be dominant. Diffuse infiltration is uncommon in PHL and indicates a poor prognosis [4]. In the USA, the nodules are usually hypoechoic or, rarely, anechoic and may resemble cysts. The absence of posterior acoustic enhancement indicates that the lesions are solid. At CT, lymphomatous nodules commonly have soft-tissue attenuation but enhance to a lesser degree than the liver parenchyma on arterial, portal venous, and delayed phase images. The lesions may demonstrate hemorrhage, necrosis, or a rim-enhancement pattern [7]. Calcification is rare in the absence of treatment. A multiphase CT study is not indicated for diagnosis of hepatic lymphoma because the lesions typically are hypovascular in all phases. At MR imaging, the nodules tend to be hypo- or isointense on T1-weighted images and moderately hyperintense on T2-weighted images, with an enhancement pattern similar to that seen at CT. In T2-weighted MR imaging, a “target” appearance, with a hyperintense poorly enhancing center and peripheral enhancement, has been described in about 15% of lesions. FDG PET/CT typically demonstrates avid hypermetabolism and is usually the imaging modality of choice for staging and for assessing treatment response [8]. Hepatologists must be aware of PHL as it may confuse with more common hepatic diseases.

Imaging findings of lymphadenopathy below the level of the renal veins, poor lesion enhancement in all contrast-enhanced phases, and vascular encasement without thrombosis favor a diagnosis of lymphoma. Imaging findings of arterial phase enhancement, delayed contrast material washout with capsular enhancement, and vascular thrombosis suggest HCC. In general, hepatic lymphomas are avidly hypermetabolic at PET, while most HCCs are not [9–12].

PHL is primarily treated with chemotherapy. R-CHOP (rituximab, cyclophosphamide, doxorubicin, vincristine, and prednisone) is the most common chemotherapy regimen for advanced diffuse large B-cell lymphoma (DLBCL). This regimen is generally given every 3 weeks for 6 to 8 cycles. Multidisciplinary approaches including surgery and radiotherapy are another options. There are reports that liver resection followed by adjuvant chemotherapy and/or radiotherapy is associated with a good prognosis [13].

## Abbreviations

PHL: Primary hepatic lymphoma
HCC: Hepatocellular carcinoma
NSAIDs: Nonsteroidal anti-inflammatory drugs
CRP: C-reactive protein
ALT: Alanine transaminase
AST: Aspartate transaminase
CEA: Carcinoembryonic antigen
FBS: Fasting blood sugar
HIV: Human immunodeficiency virus
US: Ultrasonography
MR: Magnetic resonance
CT: Computerized tomography
PET-CT: Positron emission tomography and computed tomography
